# A Case of Spontaneous Evolution of the Fœtus

**Published:** 1850-07

**Authors:** 


*A Case of Spontaneous Evolution of the Fœtus.* By J. S. Mitchell, M. D., Charleston, S. C.

*Messrs. Editors:*—As the question of "Spontaneous Evolution" is still an unsettled one, I have thought it would not be uninteresting to your readers to have the following case detailed.

Doctor Denman was, I think, among the first who boldly asserted that it was possible, under an arm and shoulder presentation, to have an unassisted termination of a case, the breech being first expelled. His idea was, that the action of the uterus, long and forcibly continued, so compacted the body of the fœtus, as to expend upon it the full force of each returning action. The body, in its doubled state, being too large to pass through the pelvis, and the uterus continuing to act with force upon the inferior extremities—they alone being movable—are driven down, and thus being forced lower, the body turns as it were upon its own axis, and the breech is expelled as in an original presentation of the same. This idea of Dr. Denman's was generally received throughout the profession, as the proper explanation, until the publication, in London, of a pamphlet, by Dr. Douglas, entitled "An Explanation of the real process of the Spontaneous Evolution of the Fœtus." In this essay, Dr. Douglass denies the position taken by Dr. Denman, and remarks that it is impossible for the uterus, while contracting, to act upon a part only of the compacted body, thus forcing it lower into the pelvis, while the other is allowed to recede into a higher position; he goes on to state that the arm, shoulder and thorax are the first expelled, and the nates and head afterwards. This latter opinion, based upon good reasons, appears now to be the one most generally adopted. By an examination of the following case, it will be evident, however, that it may happen, as stated by Dr. Denman, viz: that a case, under arm and shoulder presentation, may terminate unassisted, by spontaneous evolution, and the breech be expelled first.

On the morning of the tenth of June, 1849, I was sent for to visit Mrs. W., whom I found engaged in her third accouchment. An examination, *per vaginam,* discovered to me a head presentation; the os uteri was slightly dilated, and the pains, though trifiing, were frequent. Having other engagements of importance, I ventured to leave with the usual promise of a speedy return. It was not long after my departure, when I was again sent for, and I hurried to the bed-side of my patient. I found, on my arrival, that the head of the fætus had already passed through the os externum, and before I could make any arrangements for assisting the delivery, the remainder of the body had passed out. I quickly placed my hand upon the abdomen, with a view of securing a proper contraction of the uterus, when I discovered there a tumor of sufficient size to excite some suspicions of the presence of a second in utero. I separated the fætus from its mother, and prepared for further examination, when I discovered that the last effort of the uterus, which had expelled the first infant, had forced down the arm and shoulder of the second. This condition of things did not last long, however, for the uterus again contracting, I had the satisfaction of *seeing* the arm recede, and the nates, kindly taking its place, protruded through the vulva. I immediately seized it, determined that the capricious conduct of the infant should not again leave me in doubt and anxiety. In due time the nates was delivered, the shoulder and head following in turn. Thus ended favorably to mother and child, a case from which I had a right, under ordinary circumstances, to expect much difficulty; another evidence of the fact that a case, under arm and shoulder presentation, may, by a spontaneous evolution of the fœtus, terminate unassisted; the breech being first expelled.—*Charleston Med. Journal.*

